# Transforming acute care: a scoping review on the effectiveness, safety and implementation challenges of Hospital-at-Home models

**DOI:** 10.1136/bmjopen-2024-098411

**Published:** 2025-08-08

**Authors:** Khos Sultani, Marian Smeulers, Ralph de Vries, Barbara Maria Zonderhuis, Prabath W B Nanayakkara

**Affiliations:** 1 Acute Internal Medicine, Amsterdam UMC Locatie VUmc, Amsterdam, The Netherlands; 2 Division of Outpatient Department, Amsterdam UMC Location AMC, Amsterdam, The Netherlands; 3 Amsterdam UMC Locatie VUmc, Amsterdam, The Netherlands; 4 Surgery Department, Amsterdam UMC Locatie VUmc, Amsterdam, The Netherlands; 5 Section Acute Medicine, Department of Internal Medicine, Amsterdam Universitair Medische Centra, Amsterdam, The Netherlands

**Keywords:** Mortality, Health Services, Hospitalization

## Abstract

**Abstract:**

**Objectives:**

The hospital-at-home (HaH) model has gained traction as a viable alternative to traditional inpatient care, allowing patients to receive care in their own homes. Despite its growing popularity, there is a lack of comprehensive research addressing effectiveness, safety and factors critical to the successful implementation of HaH programmes. We conducted a scoping review to comprehensively map and summarise the evidence on both admission avoidance and early-supported discharge up until now.

**Design:**

A scoping review of randomised controlled trials (RCTs), conducted in accordance with the Preferred Reporting Items for Systematic Reviews and Meta-Analysis: extension for Scoping Reviews (PRISMA-ScR) guidelines.

**Data sources:**

Ovid MEDLINE, Embase, CINAHL and Web of Science were systematically searched up to July 2024

**Eligibility criteria for selecting studies:**

We included English-language RCTs published from 2005 onwards, involving adults (≥18 years) receiving acute care at home who would otherwise require hospital admission. Eligible studies evaluated admission avoidance or early supported discharge within HaH settings for acutely ill patients. Studies focusing on outpatient care, non-acute conditions or interventions not aligning with the widely accepted HaH definition were excluded. COVID-19-related studies were also excluded to avoid context-specific bias.

**Data extraction and synthesis:**

Two reviewers independently extracted data on study characteristics, interventions and outcomes including mortality, length of stay, escalation rates, costs and patient and caregiver satisfaction. Implementation facilitators and barriers were also collected. Discrepancies were resolved by a third reviewer. Results were synthesised descriptively in accordance with PRISMA-ScR guidelines.

**Results:**

Nine RCTs were identified. The review shows that the HaH model is at least as safe as usual care, with lower or comparable mortality rates. Length of stay varied, with some studies reporting longer stays in the HaH group due to cautious clinical practices. Cost analyses often indicate lower healthcare costs with staffing as the largest expense. Patient and caregiver satisfaction was high, but essential implementation factors were not clearly addressed.

**Conclusion:**

The HaH model represents a promising alternative to acute inpatient care for suitable patients. Future research should focus on conducting larger RCTs, expanding the range of conditions suitable for HaH. Despite favourable clinical outcomes, substantial implementation barriers remain underexplored in current RCTs. This underscores the need to identify strategies for successful implementation, including the integration of technological advancements and qualitative insights into patient and caregiver experiences.

**STRENGTHS AND LIMITATIONS OF THIS STUDY**

- Systematic searches were conducted across four major databases with a validated automatic deduplication process, ensuring an extensive and comprehensive literature search.
- Only randomised controlled trials that met a current, standardised definition of Hospital-at-Home (HaH) were included, ensuring methodological quality.
- Heterogeneity in study designs, populations and definition of HaH limited comparability across studies.
- Restricting the search to English-language publications may have introduced language bias and excluded relevant non-English studies.

## Introduction

 Over the past few decades, the concept of ‘Hospital at Home’ (HaH) has gained growing interest as an alternative to traditional hospitalisation. The HaH model aims to deliver acute hospital-level care such as intravenous therapies, monitoring of vital signs and diagnostic procedures for a wide variety of medical conditions, within the patient’s own home.^1^,^3^ During the recent COVID-19 pandemic, the interest in HaH grew immensely, emphasising the necessity and feasibility of alternative care delivery models. The ageing population, associated with an increased strain on hospitalisation capacity, has created an unprecedented urgency to find solutions that has become more pressing than ever.^4^

Furthermore, the progress in healthcare has not only led to an increased life expectancy but has also led to an increase in chronically ill patients.^3^ This shift, combined with escalating healthcare costs, underscores the importance of finding innovative, cost-efficient and effective care delivery models such as the HaH model.

The HaH model encompasses two core strategies: (1) early supported discharge and (2) admission avoidance. The concept of early supported discharge aims to facilitate the prompt discharge of hospitalised patients to the HaH while ensuring ongoing support and monitoring by a multidisciplinary team. Admission avoidance enables direct admission into the HaH through referrals from general practitioners, thereby bypassing physical contact with the hospital, or through direct admission from the emergency room without an inpatient stay.^5 6^ Both models strive to either divert or expedite conventional hospitalisation to reduce healthcare costs and hospitalisation-related complications such as nosocomial infections, delirium, thrombosis, pressure ulcers and falls. Furthermore, HaH aims to lower readmission rates and provide patient-centred care in a familiar and comfortable environment, potentially leading to higher patient satisfaction.^27^,^9^

Although HaH has been implemented for several decades, the lack of a universally accepted definition for HaH has led to heterogeneity among HaH care models. This lack of consensus has resulted in inconclusive findings regarding the effectiveness of HaH. Therefore, a generally accepted definition was formulated describing key features that define HaH. HaH is defined as a substitutive healthcare delivery model where acute hospital-level care is provided to patients in their own homes, who would otherwise be admitted to the hospital, aiming to achieve comparable or better clinical outcomes to those obtained in a traditional hospital setting.^8 10^

Multiple studies have shown the benefits of HaH in reducing adverse events associated with hospitalisation.^11 12^ A growing body of evidence indicates that both admission avoidance and early supported discharge lead to increasingly positive outcomes in terms of safety, efficacy, cost reduction and patient satisfaction.^7 9^ However, it is important to note that despite the accumulating evidence, not all studies demonstrate the presence of clear benefits of HaH interventions or contain relatively small sample sizes limiting both the strength and generalisability of the evidence. Until recently, the overall body of literature on HaH interventions has been relatively small. However, in recent years, there has been a notable surge in research and publications focused on admission avoidance and early supported discharge.^3 7 9^ Therefore, the aim of this scoping review is to comprehensively map and summarise the evidence on these two strategies for acute care up until now.

### Objectives

This scoping review maps the evidence on HaH care models that aim for either admission avoidance or early-supported discharge in acute care. Although our review exclusively includes randomised controlled trials (RCTs), the diverse nature of the HaH studies necessitated a scoping review instead of a systematic review to effectively capture the variability in study designs, patient demographics and outcomes. By focusing on RCTs, we ensure the inclusion of high-quality evidence, which allows for a reliable exploration of key concepts and factors related to HaH programmes. This approach not only enables us to clarify important definitions within the literature but also to identify critical characteristics that contribute to the effectiveness and safety of these programmes. The Population, Concept, Context framework was used to guide the development of the research questions and structure the review process. This framework supported a comprehensive overview of the current evidence and identified areas requiring further investigation.^13^

Population: Patients, aged 18 years and above, who would otherwise be admitted to the hospital but were considered suitable for receiving acute care in the HaH setting.

Concept: HaH targeted at admission avoidance or early supported discharge.

Context: Healthcare outcomes, quality of care, patient experience, cost, conditions for successful implementation.

### Research questions

1. Does admission avoidance/early-supported discharge into the HaH setting result in improved healthcare outcomes?
2. Does admission avoidance/early-supported discharge into the HaH setting result in cost reduction?
3. Does admission avoidance/early-supported discharge into the HaH setting result in improved patient experience?
4. What are the conditions for successful implementation of HaH?

## Methods

### Research design

The objective of this scoping review is to assess the impact and effect of implementing admission avoidance/early supported discharge strategies within the HaH setting and identify conditions for successful implementation. We chose a scoping review due to the diverse nature of existing studies on HaH programmes, which precludes the uniformity needed for a systematic review. Our goal is to broadly assess the literature on HaH programmes, focusing on safety and potential benefits while leveraging high-quality RCT evidence for a thorough and nuanced evaluation. This approach allows us to map key concepts and identify research gaps, providing a comprehensive overview of the field.

The Preferred Reporting Items for Systematic Reviews and Meta-Analysis: extension for Scoping Reviews (PRISMA-ScR) guideline was used to report the results of the scoping review.^14^

### Search strategy

**Figure 1:**
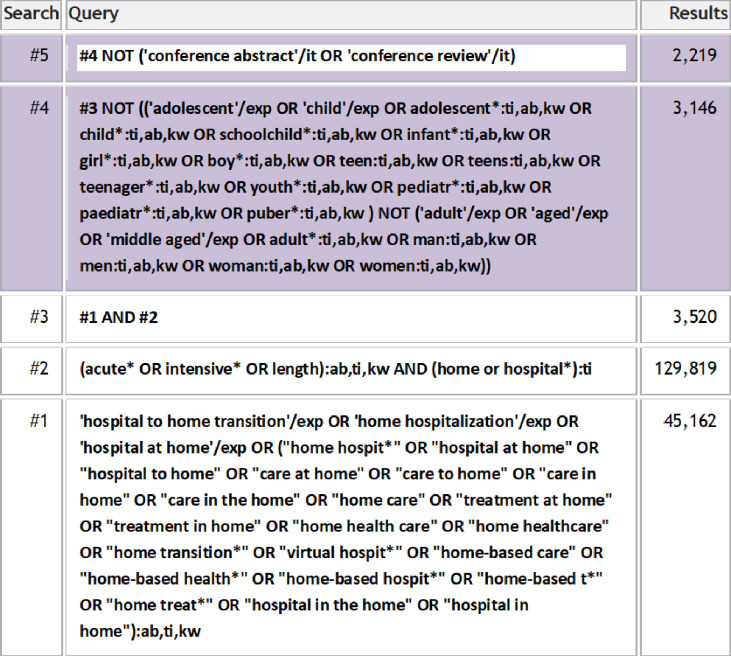
Embase search strategy.

To identify all relevant publications, we conducted systematic searches in the bibliographic databases Ovid MEDLINE, Embase.com, CINAHL (Ebsco) and Web of Science (Core Collection) from inception up to 26 July 2024, in collaboration with a medical information specialist. Figure 1 shows the Embase search strategy.

The following terms were used (including synonyms and closely related words) as index terms or free-text words:

“Hospital-based Home Care Services”, “Hospital at Home”, “Acutely ill”, “Adults”.

The references of the identified articles were searched for relevant publications. Duplicate articles were excluded by a medical information specialist using EndNote X21.0.1 (Clarivate), following the Amsterdam Efficient Deduplication method^15^ and the Bramer method.^16^ In addition, a snowballing technique was applied, whereby the reference list of included articles and relevant review papers was manually searched to identify any additional eligible articles. The full search strategies for all databases can be found in online supplemental appendix A.

### Study selection

Following the completion of the search, the retrieved articles were exported into screening management software Rayyan. Duplicate records were eliminated. The remaining articles were independently reviewed by two reviewers (KS and MS) based on predetermined inclusion and exclusion criteria. In case of discrepancies, a third reviewer (PWBN) made the final decision.

### Eligibility criteria

All RCTs related to HaH were included in this review, provided that the intervention involved either admission avoidance or early supported discharge. The focus was on RCTs that specifically targeted patients aged 18 years and above, who would otherwise be admitted to the hospital but were considered suitable for receiving acute care in the HaH setting. In November 2004, a generally accepted definition of HaH was formulated, which was further refined in 2023 to reflect the evolving nature of this care model. To ensure consistency with the current understanding of HaH, we excluded articles published before 2005, as they may not align with the standard definition.^8 10^ COVID-19-related articles were excluded to maintain a specific focus on non-pandemic-related research preventing potential bias introduced by the unique circumstances and context of COVID-19. Moreover, many HaH models implemented during the pandemic did not align with the universally accepted definition of HaH. Additionally, only English-language papers were included. We excluded articles in which interventions were delivered in outpatient care settings or did not target acutely ill patients who would otherwise be admitted to the hospital.

### Data extraction

Two reviewers carefully extracted all relevant data, focusing on study characteristics, intervention specifics and outcome measures. Outcome measures related to the effectiveness of HaH were extracted, including readmission rates, mortality, costs and length of stay (LOS). In addition, data regarding patient and caregivers’ satisfaction were also extracted to capture valuable insights into their perspectives. Facilitators and barriers to implementation were likewise extracted. The analysis of the included studies followed a systematic approach. Extracted data were used to create a synthesis to identify common trends and patterns across the studies. Furthermore, whenever feasible, subanalyses were conducted based on specific disease conditions or clinical presentations. This approach aimed to enhance comprehension regarding the impact of HaH interventions within specific medical contexts.

### Patient and public involvement

None

## Results

### Search results

**Figure 2:**
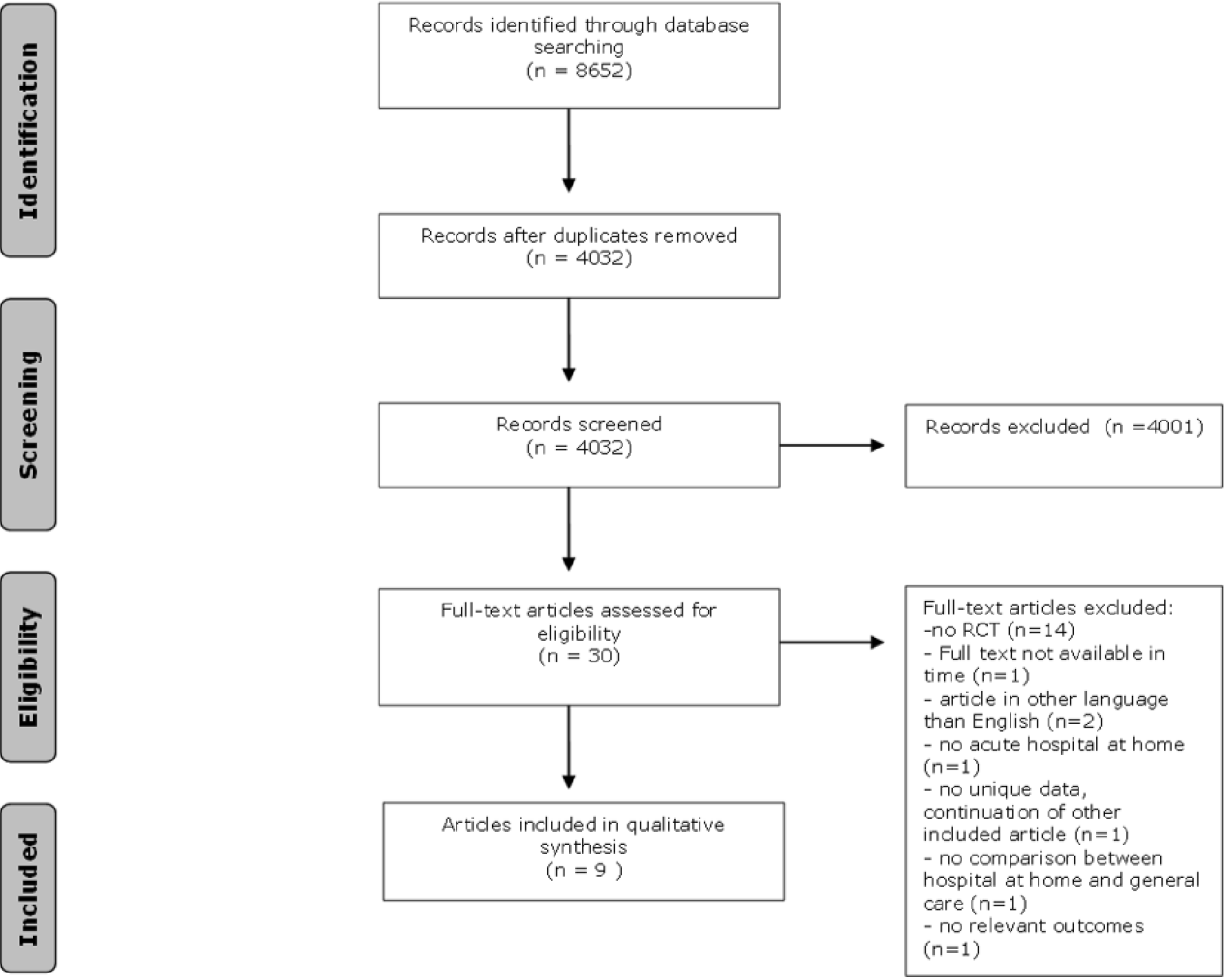
Flowchart of the search and selection procedure of studies. RCT, randomised controlled trial.

The literature search generated a total of 8652 references: 2266 in Ovid MEDLINE, 2219 in Embase.com, 2030 in CINAHL and 2137 in Web of Science. After removing duplicates of references that were selected from more than one database, 4032 references remained. The flow chart of the search and selection process is presented in figure 2.

After screening titles and abstracts, 4001 studies were excluded, resulting in a remaining 31 articles. Those full-text articles were assessed for eligibility. This resulted in the exclusion of 22 articles due to various reasons displayed in figure 1. 15 articles were excluded as they were not RCTs. One article was excluded because its endpoints did not address the research question of the scoping review. A total of nine articles remained and met our criteria.

### Study characteristics

An overview of the included studies is provided in table 1. All included papers were published in English, with geographical distribution ranging from New Zealand to Denmark. Only three studies used telemonitoring.^17^,^19^ Regarding diagnoses, the majority of studies focused on exacerbation or deterioration of chronic diseases. Specifically, seven out of nine studies included patients with exacerbations of chronic obstructive pulmonary disease (COPD).^17^,^23^ Five of the nine studies included patients with exacerbations of chronic heart failure.^12 18 19 23 24^ Six of the nine studies focused on a single diagnosis, predominantly COPD or heart failure.^1217 20^,^22 24^

**Table 1:** Study characteristics of included articles

Author (year)	Country	Use of telemonitoring	Model of HaH	Objective of the study
Diaz Lobato *et al*^21^ (2005)	Spain	No	Early-supported discharge	To evaluate the efficacy of a home hospitalisation programme for patients admitted for chronic obstructive pulmonary disease (COPD) exacerbations.
Harris *et al*^23^ (2005)	New Zealand	No	Both	To compare the safety, effectiveness, acceptability and costs of a HaH programme with usual acute hospital inpatient care.
Aimonino Ricauda *et al*^20^ (2008)	Italy	No	Both	To evaluate hospital readmission rates and mortality at 6-month follow-up in selected elderly patients with acute exacerbation of COPD.
Mendoza *et al*^24^ (2009)	Spain	No	Admission avoidance	To assess the effectiveness of HaH compared with inpatient hospital care on the combined outcome of mortality, heart failure re-admission or other cardiovascular event (stroke, acute coronary syndrome and coronary revascularisation) as well as the evolution of functional status and quality of life during the index episode and after 1 year of follow-up. The second aim was to compare the health expenditure on each type of care during the initial episode and after 1 year.
Tibaldi *et al*^12^ (2009)	Italy	No	Both	To evaluate the feasibility and effectiveness of a physician-led HaH service for selected elderly patients with acute decompensation of chronic heart failure.
Jakobsen *et al*^17^ (2015)	Denmark, Copenhagen	Yes	Early-supported discharge	To investigate a telemedicine-based treatment solution for patients with acute exacerbation of COPD at home as compared with conventional hospital treatment measured according to first treatment failure, which is defined as readmission due to COPD within 30 days after discharge.
Echevarria *et al*^22^ (2018)	UK	No	Early-supported discharge	To conduct an economic evaluation (cost-effectiveness analysis) comparing HaH with usual care (UC) in patients admitted with a low-risk ECOPD selected by DECAF score. The trial examined whether, within a non-inferiority limit of £150, the total health and social care costs up to 90 days associated with HAH are the same or less than those from UC.
Levine *et al*^18^ (2018)	Boston, USA	Yes	Admission avoidance	To determine if home hospital care reduces costs while maintaining quality, safety and patient experience compared with inpatient hospital care.
Levine *et al*^19^ (2020)	Boston, USA	Yes	Admission avoidance	To compare outcomes (total direct cost, healthcare use, physical activity) of HaH versus usual hospital care for patients requiring admission.

DECAF, Dyspnoea, Eosinopenia, Consolidation, Acidaemia, and atrial Fibrillation (a prognostic score for patients with acute exacerbation of COPD); ECOPD, Exacerbation chronic obstructive pulmonary disease ; HaH, hospital-at-home.

The number of participants in the studies ranged from 20 to 285, with a mean of 98.8 participants. Three out of the nine studies specifically aimed to include patients over the age of 65.^12 20 24^ The mean age of study participants ranged from 65 to 82.2 years in the HaH group and from 60 to 80.1 years in the usual care group. Gender distribution was generally balanced across the studies, with only one study not reporting the gender breakdown.^23^

### Outcomes

Primary endpoints were mostly focused on the safety and efficacy of the HaH model, although in some articles, costs were the primary endpoint.^18 19 22^ Secondary outcomes displayed some overlap but varied in the time frames used for measurement. An overview is given in onlinesupplemental tables 2  3. Other findings are shown in online supplemental table 4.

#### Mortality

Mortality during admission was reported in seven articles.^1218^,^20 22^ All of these articles indicated that the HaH model had mortality rates that were lower or comparable to those of usual care. Two articles did not report mortality during the acute care period but provided overall mortality from admission through follow-up.^19 22^ Mortality rates during the acute care period ranged from 0% to 8.7%.^1218^,^20 22^

#### Length of stay

In four out of nine articles,^12 20 23 24^ the LOS was significantly longer in the HaH group compared with usual care, as displayed in online supplemental table 2. In contrast, two articles^17 18^ found no significant difference, and two did not report p values.^19 22^ One study^21^ noted a significantly shorter LOS for the HaH group compared with usual care.

#### Escalation rates

Escalation rates are not mentioned in two articles.^22 23^ Three articles reported no escalations during admission.^18 19 24^ Escalation rates in the remaining articles range from 5% to 30.8%.^12 17 20 21^ The escalation rate of 30.8% primarily stemmed from the necessity for diagnostics, accounting for 21.1% of the cases within this group.^20^ Other common reasons for escalations include caregiver problems, clinical deterioration or complications related to treatment or disease.

#### Costs

Costs were reported as an outcome in seven^1218^,^20 22^ of the nine studies, with variations in length of measurement: including costs over a 6-month period and costs incurred from admission to the end of the hospital stay.

All of these studies assessed the costs of the acute care period. Some studies additionally measured healthcare costs for the admission period followed by either 30-day costs,^18^ 90-day costs^22^ or annual costs.^24^ Six studies^1218^,^20 22 24^ reported significantly lower costs, as presented in online supplemental table 2, for the acute care period in the HaH model compared with usual care. One article stated that the cost of an admission to the HaH model was significantly higher due to a longer stay or more bed days. However, if capacity were to increase, the cost should not be significantly different from that of usual care.^23^

Cost analyses in multiple studies indicated that the majority of expenses within the HaH model were attributed to staff costs.^20^ The reduction in costs within the HaH model was due to the lower average cost of stay, despite the average stay being longer than inpatient hospital care.^19 22 24^ Furthermore, the expenditures on investigations and consumables were lower.^18 19 22 24^

#### Patient experience

Patient experience was evaluated in five articles.^17^,^2023^ All these studies reported high levels of satisfaction among patients and/or caregivers. Additionally, satisfaction levels among nurses and doctors are consistently reported as being high.

#### Implementation factors

None of the reviewed articles explicitly identified key factors for successful implementation of HaH programmes. However, one study^17^ did highlight a significant barrier to participation: many non-participants were not deterred by the technology itself but found the logistics of being moved between home and the hospital within the same day too burdensome. This suggests that patients might prefer the integration of telehealth prior to hospitalisation, which could be a critical consideration for improving implementation strategies.

## Discussion

The purpose of this scoping review was to evaluate the impact and effectiveness of implementing a HaH model. Additionally, we sought to identify the key conditions for its successful implementation. During the review process, it became evident that there is a scarcity of well-designed RCTs in this field which aligns with previous findings in the literature.^7 9^

The majority of articles we reviewed were pilot or feasibility studies. This trend is primarily because globally HaH programmes are still developing or evolving, driven by technological advancements such as telemonitoring. The COVID-19 pandemic has contributed to recognising the benefits and added value that telemonitoring can provide, which can be helpful in establishing a sustainable HaH model.^25^

Our study indicates that a HaH programme is at least as safe and effective as usual care.^7 9^ All included articles documented lower or comparable mortality rates compared with usual care. The LOS within the HaH programme was generally longer, which could be partly attributed to the caution exercised by healthcare providers due to the novelty of the concept and their limited experience with it. Although a longer LOS generally increases costs, one study^23^ suggests that with expanded capacity, these costs may not differ significantly from usual care. The higher costs of the HaH programme can be attributed to its intensive care model, which included full-time medical staff, round-the-clock nursing and 24-hour home support. By integrating such services into existing administrative structures, the costs per patient could potentially be reduced through shared resources and overheads. Moreover, most studies^1218^,^20 22 24^ report lower costs despite the extended LOS in HaH programmes.

We also aimed to examine the conditions for successful implementation. However, the RCTs reviewed provided little to no information on this aspect. To investigate this further, future research may need to focus on qualitative studies that dedicate more attention to the factors influencing successful implementation.

Qualitative findings suggest that factors such as early stakeholder engagement, clear communication strategies, adequate staff training and addressing safety concerns are critical for successful HaH implementation. Additionally, multidisciplinary care teams, accessibility to emergency care, provider training and robust referral criteria are essential to ensuring successful outcomes. Future research should explore these aspects further, especially in diverse healthcare settings such as rural settings and lower-income countries.

It is also crucial to examine the impact on caregivers and ensure patient and caregiver preferences are considered in the decision-making process.^26 27^

There was considerable variation in escalation rates, partly due to differing definitions and interpretations of what constitutes an escalation. For example, one study^20^ considers the need for hospital-based diagnostics as an escalation, whereas other studies might not. Similarly, regarding LOS, some studies count only the physical hospital bed days, while others include the entire duration from hospital admission to discharge from the HaH setting. In our view, performing diagnostics requiring transport to the hospital constitutes an escalation because it necessitates hospital transportation and prevents necessary care from being delivered at home. The same applies if informal caregiver support fails, leading to escalation back to the hospital. An escalation does not necessarily pertain solely to patient deterioration but rather to deviations from the planned protocol that require physical hospital attendance. Regarding LOS, it encompasses the total duration in days of both hospital and home stay. Throughout this period, the healthcare provider assumes responsibility for the patient’s care, regardless of whether the care is delivered in a physical ward or a virtual setting.

Furthermore, it is important to note that the concept and definition of HaH still show considerable variation across the world, which was also stated earlier by Levine *et al.* Partially, this variation stems from disparities in healthcare systems and their organisational structures. What is referred to as HaH in one country might not meet the criteria for HaH in another country, which further complicates comparable research.

Having a clear and uniform definition of ‘Hospital-at-Home’ is crucial for conducting generalisable research and evaluating its effectiveness and safety reliably. It also supports research comparability, consistent assessment of effectiveness and safety, policy development, clinical implementation, quality monitoring and improves communication among stakeholders. Despite having a clear definition of HaH, the search and inclusion process for this study was particularly challenging. The diversity of study designs, varying interpretations of the HaH model and inconsistent reporting on relevant outcomes made it difficult to identify studies that aligned with our criteria. This required extensive screening to ensure the inclusion of studies that truly reflected the scope of this review.

Many articles have provided evidence supporting the feasibility and benefits of HaH models. However, there remains a shortage of large RCTs that assess the true effect size, impact on patient-related outcomes and consequently our healthcare systems. The existing RCTs are characterised by small sample sizes and diverse patient populations, which not only restrict the generalisability of the findings but also impair the detection of clinically significant differences and condition-specific outcomes. Furthermore, current research pays limited attention to the implementation, facilitators and barriers of HaH programmes. HaH is a complex intervention that necessitates a mixed methods approach for thorough evaluation. Integrating quantitative RCT data with qualitative insights can elucidate how HaH works, its impact mechanisms and how to tailor interventions to patient needs. Given the limited examples and guidance on such techniques, future research will benefit from clear directions on applying and assessing mixed methods in HaH studies. Additionally, the use of new technological tools like telemonitoring is underexplored, despite their potential to enhance care efficiency and (cost)effectiveness.

It is understandable that chronic diseases such as COPD and congestive heart failure are the primary focus of extensive research, due to their significant impact on the healthcare crisis and the high costs associated with their management and treatment. However, this narrow focus limits the generalisability of findings. Other acute and complex conditions, such as infections, postoperative recovery and oncology, may also benefit from HaH interventions. Future research is needed to explore the feasibility and effectiveness of HaH in these less studied populations.

We observe that costs for staff are often the largest expense in HaH care.^20^ Advances in technologies such as telemonitoring may reduce the need for extensive staffing. Additionally, new technological advances have made it possible to deliver more complex care,^28^ such as chemotherapy, remotely, potentially leading to further cost and staff savings. For instance, Nørskov *et al*^13 29^ demonstrated that this approach was feasible and safe for patients with acute myeloid leukaemia or high-risk myelodysplastic syndrome during induction and consolidation treatments. Patients used a portable, programmable CADD (Computerized Ambulatory Delivery Device) pump for chemotherapy at home, supported by a patient educational programme. This programme trained patients and caregivers on managing the central venous catheter, using the infusion pump and responding to pump alarms, ensuring safe and effective home-based care. Technological advancements like these could play a crucial role in addressing the global healthcare crisis, where managing costs and staffing are vital considerations.

Continuously emphasised is the pivotal role of patient inclusion and selection in HaH programmes. Enhanced filtering for adequate selection of patients for HaH programmes might correlate positively with patient outcomes, as well as escalation rates and costs.^22^ Furthermore, the process of screening and identifying HaH candidates appears notably time-consuming, potentially benefitting from the integration of artificial intelligence. Alongside objective criteria for patient selection, a deeper understanding of the reasons behind patient and caregiver participation or non-participation is also needed.

Qualitative research into patient and caregiver participation to HaH programmes is imperative in elucidating reasons for non-participation in HaH programmes. Such insights could foster enhanced participation rates and programme effectiveness.

### Limitations

This scoping review has several limitations. First, the scarcity of large, high-quality RCTs reduces the overall strength and generalisability of the findings. Additionally, the small sample sizes of the included studies limit the ability to detect clinically significant differences. The heterogeneity in study designs, patient populations and definitions of HaH complicates comparisons across studies.

We limited our search to English-language articles to ensure accurate data extraction and consistent interpretation among the two reviewers (KS and MS). Given the considerable heterogeneity in study designs and international variation in the definition of HaH, the inclusion of non-English studies could have further complicated interpretation and comparability. While this may have introduced language bias, restricting to English is a common and pragmatic choice in many published scoping reviews. Moreover, few studies explicitly address the conditions for successful implementation, leaving gaps in understanding real-world applicability. Finally, the focus on chronic diseases like COPD and heart failure overlooks other potential conditions that could benefit from HaH interventions. Additionally, by limiting our inclusion to RCTs and applying very narrow selection criteria, we may have missed robust, pragmatic studies using alternative designs, such as matched-pairs. These studies could have offered valuable evidence on the effectiveness and real-world applicability of HaH interventions.

## Conclusion

The HaH model represents a promising alternative to inpatient care for suitable patients associated with comparable or even better clinical outcomes. Mortality rates are comparable or even lower. LOS varied among studies, with some studies even reporting longer stays in the HaH group due to cautious clinical practices. Cost analysis often indicates lower healthcare costs with staffing as the largest expense. It has the potential to address rising healthcare costs and alleviate the ongoing strain on the healthcare system, offering a promising outlook for the future. Future research should focus on conducting larger RCTs and expanding the range of conditions suitable for HaH. Furthermore, it is important to identify strategies for successful implementation, including the integration of technological advancements and qualitative insights into patient and caregiver experiences.

## Supplementary material

10.1136/bmjopen-2024-098411 online supplemental file 1

10.1136/bmjopen-2024-098411 online supplemental file 2

10.1136/bmjopen-2024-098411 online supplemental file 3

10.1136/bmjopen-2024-098411 online supplemental file 4

## Data Availability

Data are available in a public, open access repository.
