# Supplementary material for: Transforming acute care: a scoping review on the effectiveness, safety and implementation challenges of Hospital-at-Home models
Source: BMJ Open. 2025 Aug 8;15(8):e098411. doi: 10.1136/bmjopen-2024-098411 (PMC12336546; doi:10.1136/bmjopen-2024-098411)
Supplement: online supplemental file 1 [file bmjopen-15-8-s001.docx]

**Appendix A**

**Ovid Medline Search Results – 26 Jul 2024**

| **Search** | **Query** | **Results** |
| --- | --- | --- |
| #4 | **3 not ((exp Adolescent/ or exp Child/ or exp Infant/ or (adolescen* or child* or schoolchild* or infant* or girl* or boy or boys or teen or teens or teenager* or youth* or pediatr* or paediatr* or puber*).ab,ti,kf.) not (exp Adult/ or (adult* or man or men or woman or women).ab,ti,kf.))** | 2,266 |
| #3 | **1 and 2** | 2,557 |
| #2 | **(acute* or intensive* or length).ab,ti,kf. and (home or hospital*).ti.** | 79,750 |
| #1 | **exp "Home Care Services, Hospital-Based"/ or exp "Hospital to Home Transition"/ or ("home hospit*" or "hospital at home" or "hospital to home" or "care at home" or "care to home" or "care in home" or "care in the home" or "home care" or "treatment at home" or "treatment in home" or "home health care" or "home healthcare" or "home transition*" or "virtual hospit*" or "home-based care" or "home-based health*" or "home-based hospit*" or "home-based t*" or "home treat*" or "hospital in the home" or "hospital in home").ab,ti,kf.** | 37,854 |

**Embase.com Search Results – 26 Jul 2024**

| **Search** | **Query** | **Results** |
| --- | --- | --- |
| #5 | **#4 NOT ('conference abstract'/it OR 'conference review'/it)** | 2,219 |
| #4 | **#3 NOT (('adolescent'/exp OR 'child'/exp OR adolescent*:ti,ab,kw OR child*:ti,ab,kw OR schoolchild*:ti,ab,kw OR infant*:ti,ab,kw OR girl*:ti,ab,kw OR boy*:ti,ab,kw OR teen:ti,ab,kw OR teens:ti,ab,kw OR teenager*:ti,ab,kw OR youth*:ti,ab,kw OR pediatr*:ti,ab,kw OR paediatr*:ti,ab,kw OR puber*:ti,ab,kw ) NOT ('adult'/exp OR 'aged'/exp OR 'middle aged'/exp OR adult*:ti,ab,kw OR man:ti,ab,kw OR men:ti,ab,kw OR woman:ti,ab,kw OR women:ti,ab,kw))** | 3,146 |
| #3 | **#1 AND #2** | 3,520 |
| #2 | **(acute* OR intensive* OR length):ab,ti,kw AND (home or hospital*):ti** | 129,819 |
| #1 | **'hospital to home transition'/exp OR 'home hospitalization'/exp OR 'hospital at home'/exp OR ("home hospit*" OR "hospital at home" OR "hospital to home" OR "care at home" OR "care to home" OR "care in home" OR "care in the home" OR "home care" OR "treatment at home" OR "treatment in home" OR "home health care" OR "home healthcare" OR "home transition*" OR "virtual hospit*" OR "home-based care" OR "home-based health*" OR "home-based hospit*" OR "home-based t*" OR "home treat*" OR "hospital in the home" OR "hospital in home"):ab,ti,kw** | 45,162 |

**CINAHL (Ebsco) Search Results – 26 Jul 2024**

| **Search** | **Query** | **Results** |
| --- | --- | --- |
| S4 | **S3 NOT (TI (("adolescen*" OR "child*" OR "schoolchild*" OR "infant*" OR "girl*" OR "boy*" OR "teen" OR "teens" OR "teenager*" OR "youth*" OR "pediatr*" OR "paediatr*" OR "puber*") NOT ("adult*" OR "man" OR "men" OR "woman" OR "women")) OR AB (("adolescen*" OR "child*" OR "schoolchild*" OR "infant*" OR "girl*" OR "boy*" OR "teen" OR "teens" OR "teenager*" OR "youth*" OR "pediatr*" OR "paediatr*" OR "puber*") NOT ("adult*" OR "man" OR "men" OR "woman" OR "women")))** | 2,030 |
| S3 | **S1 AND S2** | 2,438 |
| S2 | **(TI ("acute*" OR "intensive*" OR "length") OR AB ("acute*" OR "intensive*" OR "length")) AND TI ("home" OR "hospital*")** | 33,333 |
| S1 | **(MH "Home Health Care+") OR TI ("home hospit*" OR "hospital at home" OR "hospital to home" OR "care at home" OR "care to home" OR "care in home" OR "care in the home" OR "home care" OR "treatment at home" OR "treatment in home" OR "home health care" OR "home healthcare" OR "home transition*" OR "virtual hospit*" OR "home-based care" OR "home-based health*" OR "home-based hospit*" OR "home-based t*" OR "home treat*" OR "hospital in the home" OR "hospital in home") OR AB ("home hospit*" OR "hospital at home" OR "hospital to home" OR "care at home" OR "care to home" OR "care in home" OR "care in the home" OR "home care" OR "treatment at home" OR "treatment in home" OR "home health care" OR "home healthcare" OR "home transition*" OR "virtual hospit*" OR "home-based care" OR "home-based health*" OR "home-based hospit*" OR "home-based t*" OR "home treat*" OR "hospital in the home" OR "hospital in home")** | 64,571 |

**Web of Science (Core Collection) Search Results – 26 Jul 2024**

| **Search** | **Query** | **Results** |
| --- | --- | --- |
| #4 | **#3 NOT TS=(("adolescen*" OR "child*" OR "schoolchild*" OR "infant*" OR "girl*" OR "boy*" OR "teen" OR "teens" OR "teenager*" OR "youth*" OR "pediatr*" OR "paediatr*" OR "puber*") NOT ("adult*" OR "man" OR "men" OR "woman" OR "women"))** | 2,137 |
| #3 | **#1 AND #2** | 2,469 |
| #2 | **TS=("acute*" OR "intensive*" OR "length") AND TI=("home" OR "hospital*")** | 91,694 |
| #1 | **TS=("home hospit*" OR "hospital at home" OR "hospital to home" OR "care at home" OR "care to home" OR "care in home" OR "care in the home" OR "home care" OR "treatment at home" OR "treatment in home" OR "home health care" OR "home healthcare" OR "home transition*" OR "virtual hospit*" OR "home-based care" OR "home-based health*" OR "home-based hospit*" OR "home-based t*" OR "home treat*" OR "hospital in the home" OR "hospital in home")** | 36,213 |
