# Supplementary material for: Transforming acute care: a scoping review on the effectiveness, safety and implementation challenges of Hospital-at-Home models
Source: BMJ Open. 2025 Aug 8;15(8):e098411. doi: 10.1136/bmjopen-2024-098411 (PMC12336546; doi:10.1136/bmjopen-2024-098411)
Supplement: online supplemental file 2 [file bmjopen-15-8-s002.docx]

**Supplementary table 1.**  Endpoints and conclusions

| Author (year) | No. Participants | Study population | Endpoints | Conclusion |
| --- | --- | --- | --- | --- |
| Diaz  (2005) | 40  HaH: 20  UC: 20 | <85 years and exacerbation of COPD | Primary:  Therapeutic failures  Secondary:  number of calls for consultation or medical assistance, number of relapses at 1 month of follow up, extent of smoking cessation among smokers, and the number of hospital admissions saved in the HH | Our results show that a hospital-supervised HH program including the participation of pneumologists and nursing staff allows for the recovery of patients hospitalized for exacerbation of COPD who have stable symptoms and arterial blood gases with no increase in the rate of readmission, relapse, or therapeutic failure. |
| Harris  (2005) | 285  HaH: 143  UC: 142 | ≥ 55 years and an acute medical problem | Primary:  Functional independence measure *FIM), MMSE, IADL by OARS,   Secondary:  self-reported recovery, health status as assessed by the SF-36 (acute form), withdrawal from the study, readmissions to the hospital, falls, bladder problems, bowel problems, confusion, admission to an institution for permanent care, mortality  Costs Patient satisfaction | The hospital-at-home programme was found to be more acceptable and as effective and safe as inpatient care. While caring for patients at home was significantly more costly than standard inpatient care, this was largely due to the hospital-at-home programme not operating at full capacity. |
| Aimonino (2008) | 104  HaH: 52  UC: 52 | ≥ 75 years and an acute exacerbation of COPD | Primary:  Hospital readmission and mortality rates at 6 months,   Secondary:  depression status, functional status, cognitive status, quality of life, nutritional status Costs  Patient satisfaction | Physician-led substitutive hospital-at home care as an alternative to inpatient care for elderly patients with acute exacerbations of COPD is associated with a substantial reduction in the risk of hospital readmission at 6 months, lower healthcare costs, and better quality of life. |
| Mendoza  (2009) | 71  HaH: 37  UC: 34 | ≥ 65 years and acute decompensation of chronic heart failure | Mortality, readmission, cardiovascular event (stroke, acute coronary syndrome, and coronary revascularization), functional status, quality of life during the index episode and after 1 year of follow-up   Costs during the initial episode and after 1 year. | Hospital at home care allows an important reduction in the costs during the index episode compared with hospital care, whilst maintaining similar outcomes with respect to cardiovascular mortality and morbidity and quality of life at 1 year follow-up. |
| Tibaldi  (2009) | 101  HaH: 48  UC: 53 | ≥ 75 years and acute decompensation of chronic heart failure | Primary:  Mortality at 6 months  Secondary:  Morbidity: infections, delirium, bed scores, deep vein thrombosis and falls, Admissions to a nursing home, and subsequent hospital admissions related to any cause  Quality of life  Costs | Substitutive hospital-at-home care is a viable alternative to traditional hospital inpatient care for elderly patients with acutely decompensated CHF. This type of care demonstrated clinical feasibility and efficacy in comparison with its alternative. |
| Jakobsen  (2015) | 57  HaH: 29  UC: 28 | ≥ 45 years and admitted for acute exacerbation of COPD | Primary:  Treatment failure defined as readmission due to exacerbation in COPD within 30 days.  Secondary:  Mortality, need of manual or mechanical ventilation or NIV, physiological measures, length of hospitalization, health-related quality of life, user satisfaction (patient and health professional), adverse events, and healthcare costs. | Whether home-based telehealth hospitalization is noninferior to conventional hospitalization requires further investigation. The results indicate that a subgroup of patients with severe COPD can be treated for acute exacerbation at home using telehealth, without the physical presence of health professionals and with a proper organizational ‘‘back-up.’’ |
| Echevarria  (2018) | 120  HaH: 62  UC: 58 | ≥ 35 years and acute exacerbation of COPD with a low-risk ECOPD (DECAF 0 or 1) | Primary:  Total cost of health and formal social care over 90 days from presentation costed from a UK health and social care perspective  Secondary:  Survival, readmission rate, total bed days over 90 days, length of hospital stay (LOHS), and  cost-effectiveness, using the Euro Quality of life instrument (EQ-5D-5L) quality-adjusted life year (QALY) measured at baseline, 14 and 90 days, patient preference for HAH or UC, COPD exacerbations, Hospital Anxiety and Depression Scale scores (HADS), and COPD Assessment Tool (CAT) scores | HAH selected by low-risk DECAF score was safe, clinically effective, cost-effective, and preferred by most patients. Compared with earlier models, selection is simpler and approximately twice as many patients are eligible. The introduction of DECAF was associated with a fall in UC length of stay without adverse outcome, supporting use of DECAF to direct early discharge. |
| Levine (2018) | 20  HaH: 9  UC: 11 | ≥ 18 years and a primary diagnosis of any infection, heart failure exacerbation, COPD exacerbation, or asthma exacerbation | Primary:  direct cost of the acute care episode.   Secondary: included utilization, 30-day cost, physical activity, and patient experience. | The use of substitutive home hospitalization compared to in-hospital usual care reduced cost and utilization and improved physical activity. No significant differences in quality, safety, and patient experience were noted, with more definitive results awaiting a larger trial. |
| Levine (2020) | 91  HaH: 43  UC: 48 | ≥ 18 years and a primary diagnosis of any infection, heart failure exacerbation, COPD exacerbation, or asthma exacerbation | Primary:  the total direct cost of the acute care episode (sum of costs for nonphysician labour, supplies, medications, and diagnostic tests).   Secondary:  health care use, physical activity during the acute care episode and at 30 days, patient experience, safety, and quality during the acute care episode | Substitutive home hospitalization reduced cost, health care use, and readmissions while increasing physical activity compared with usual hospital care. |
