# Supplementary material for: Transforming acute care: a scoping review on the effectiveness, safety and implementation challenges of Hospital-at-Home models
Source: BMJ Open. 2025 Aug 8;15(8):e098411. doi: 10.1136/bmjopen-2024-098411 (PMC12336546; doi:10.1136/bmjopen-2024-098411)
Supplement: online supplemental file 3 [file bmjopen-15-8-s003.docx]

**Supplementary table 2.**  Outcomes

| Author (year) | Mortality during admission | Length of stay | Escalation | Patient satisfaction | Costs |
| --- | --- | --- | --- | --- | --- |
| Diaz  (2005) | Not specifically reported  One patient in the usual care group died | HaH group:  9.2 days (4 days in the hospital and 5 at home) Usual care:  12.2 days P = <0.05 | 1 (5%) patient due to nonspecific abdominal pain | Not reported | Not reported |
| Harris (2005) | No difference in mortality | HAH group:  8.8 days Usual care: 5.7 days P= 0.01 | Not reported for the acute phase | HaH group: Overall satisfaction as very good or excellent (83%)  Usual care: Overall satisfaction as very good or excellent (72.3%) | The mean cost per patient was almost twice for patients treated at home (NZ$6524) as for standard hospital care (NZ$3525). A sensitivity analysis indicated that, if the service providing care in the home had been operating at full capacity, the mean cost per patient episode would have been similar for both modes of care. |
| Aimonino (2008) | Upon discharge no difference in mortality (9 deaths, 8.7%, in total) | HAH group:  15.5 days Usual care:  11.0 days P=0.01  *Patients managed in the GHHS had a longer mean length of stay than those cared for in the GMW (15.5 9.5 vs 11.0 7.9 days, P5.010).* | 16 (30.8%) escalations:  11 (21.1%) due to necessity for diagnostics 3 (5.8%) due to caregiver’s health problems  2 (3.8%) due to cilinical deterioration and the need for admission to the ICU | HaH group: Satisfaction at discharge was very good or excellent (94%)  Usual care: Satisfaction at discharge was very good or excellent (88%) | *On a cost per patient per day basis, GHHS costs were lower than costs in GMW ($101.4 ± 61.3 vs $151.7 ± 96.4, P < 0.002).* |
| Mendoza (2019) | No deaths occurred during initial admission. | HaH group:  10.9 days (SD:5.9 days) Usual care:  7.9 days (SD:3.0 days)  P=0.01 | No escalations | Not reported | The average cost of the initial admission was 4502+2153E in IHC and 2541+1334E in HaH (P < 0.001). During 12 months of follow-up, the average expenditure was 4619+7679E and 3425+4948E (P = 0.83) respectively.  The reduction of costs during the index admission in our study was 44%, related mainly to the lower expenditure on hospital stays. Furthermore, there were also savings in the HaH group due to the lower rate of investigations and use of consumables |
| Tibaldi (2009) | At discharge 3 (3%) deaths each | HaH group:  20.7 days (SD: 6.9 days) Usual care:  11.6 days (SD: 10.7 days) P=0.001 | 4 (8%) escalations   1( 2%) due to fall 1 (2%) due to intestinal bleeding  1 (2%) due to minor stroke  1 (2%) due to health problems caregiver | Not reported | The mean total cost was €1820.92 (US $2604.46) for each patient treated at home and €2116.89 (US $3027.78) for patients treated in the hospital (P< 0.001).  On a costper- patient-per-day basis, hospital-at-home costs were on average €110.98 (US $158.73) compared with (US $401.37) €280.62 for in-hospital patients. |
| Jacobsen (2015) | no mortality reported for the acute phase | HaH group:  5 (17,2%) patients were hospitalized for more than 5 days Usual care:  8 (28,6%) patients were hospitalized for more than 5 days P=0.48 | 3 (10.3%) escalations  1 (3.4%) due to technical failure of wireless broadband technology in the home 1 (3.4%) due to hyponatremia 1 (3.4%) due to severe dyspnea | Not reported | Not reported |
| Echevarria (2018) | There were no deaths in the acute period (within 14 days) in either arm. | HaH group:   Length of stay during the index admission in UC was only 3 days, which was 2 days shorter than expected | Not reported | Not reported | Mean 90-day costs were £1016 lower in HAH, but the one-sided 95% CI crossed the non-inferiority limit of £150 (CI −2343 to 312). Savings were primarily due to reduced hospital bed days: HAH=1 (IQR 1–7), UC=5 (IQR 2–12) (P=0.001).  Based on quality-adjusted life years, the probability of HAH being cost-effective was 90%. |
| Levine (2018) | There were no deaths | HaH group: 3 days Usual care: 3 days (p=0.8) | No escalations | HaH group: Reported high global satisfaction with care (median score, 10 of 10) Would recommend their acute care experience (4 of 4; interquartile range, 0)  Usual care: Reported high global satisfaction with care (median score, 10 of 10) Would recommend their acute care experience (4 of 4; interquartile range, 0) | Median direct cost of the acute care episode for home patients was 52% (IQR, 28%; p = 0.05) lower than for control patients.   Median direct cost for the acute care plus 30-day post-discharge period for home patients was 67% (IQR, 77%; p < 0.01) lower, with trends toward less use of home-care services (22% vs. 55%; p = 0.08) and fewer readmissions (11% vs. 36%; p=0.32). |
| Levine (2020) | There were no deaths | HaH group:  4.5 days (CI: 3.9 to 5.0 days)  Usual care:  3.8 days (CI: 3.3 to 4.4 days) | No escalations | HaH group: Reported high global satisfaction with care (median score, 10 of 10) Would recommend their acute care experience (4 of 4; interquartile range, 0)  Usual care: Reported high global satisfaction with care (median score, 10 of 10) Would recommend their acute care experience (4 of 4; interquartile range, 0) | The adjusted mean cost of the acute care episode was 38% (95% CI, 24% to 49%) lower for home patients than control patients.  Mean unadjusted cost of the acute care episode was 41% lower for home patients than control patients (P < 0.001). Adjusted mean cost was 38% lower (95% CI, 24% to 49% lower; P < 0.001) |
