# Supplementary material for: Transforming acute care: a scoping review on the effectiveness, safety and implementation challenges of Hospital-at-Home models
Source: BMJ Open. 2025 Aug 8;15(8):e098411. doi: 10.1136/bmjopen-2024-098411 (PMC12336546; doi:10.1136/bmjopen-2024-098411)
Supplement: online supplemental file 4 [file bmjopen-15-8-s004.docx]

**Supplementary table 3.** Other findings

| Author (year) | Other findings |
| --- | --- |
| Diaz  (2005) | No significant differences in the number of calls for consultation or medical assistance There were differences in the number of calls for emergency assistance (4 calls in the conventional hospitalization group compared to none in the HH group).  Significant differences in the number of relapses during the 1-month follow-up period (5 patients in the conventional hospitalization group presented signs and symptoms of exacerbation during this period compared to none in the patients treated at home). There was 1 case of therapeutic failure in each group.   There were no differences in smoking habits between the 2 groups upon admission (8 patients in the HH group and 10 in the control group were smokers), although at 1-month follow up a greater number of patients in the HH group had remained abstinent: 2 were still smoking in the HH group and 8 in the control group (P<.05). |
| Harris (2005) | No statistically significant differences between the two groups for any of the secondary health outcomes  Readmissions:  HAH; Not readmitted 99 (69.2%)  Hospital care: Not readmitted 109 (76.8%)  Although the number of readmissions to hospital was higher in the hospital-at-home group in the first 10 days after randomization, there was no difference between the two groups beyond 10 days. |
| Aimonino (2008) | Only GHHS patients experienced improvements in depression and quality-of-life scores.   There was a lower incidence of hospital readmissions for GHHS patients than for GMW patients at 6- month follow-up (42% vs 87%, P < 0.001).  Cumulative mortality at 6 months was 20.2% in the total sample, without significant differences between the two study groups. |
| Mendoza (2019) | Clinical outcomes were similar after initial admission and also after the 12 months of follow-up. Changes in functional status and health-related quality of life over the follow-up period were not significantly different.   During the 12 months after the intervention, five patients died, three from the IHC group and two from the HaH group (P = 0.6). |
| Tibaldi (2009) | Only the GHHS patients experienced improvements in depression, nutritional status, and quality-of-life scores.  The number of subsequent hospital admissions was not statistically different in the 2 groups, but the mean (SD) time to first additional admission was longer for the GHHS patients (84.3 [22.2] days vs 69.8[36.2] days, P=.02).   Patient mortality at 6 months was 15% in the total sample, without significant differences between the 2 settings of care |
| Jacobsen (2015) | The scores of CCQ, SGRQ and EQ-5D improved in both groups over time within the first 30 days after discharge, but the improvement was not significantly different between groups  No participants died within 30 days after discharge. In the control group four participants died during 6 months of follow-up compared with three participants in the intervention group. Overall survival was evaluated based on registry data for June 2013. This showed a survival probability 2 years after randomization of 59.2% (40.2–78.1%) in the control group and of 82.8% (69.0–96.5%) in the intervention group (by log-rank test, p = 0.053)  Cox regression showed no significant difference between the readmission rates (hazard ratio = 2.01; 95% confidence interval, 0.71– 5.71). |
| Echevarria (2018) | Length of stay during the index admission in UC was only 3 days, which was 2 days shorter than expected. There was one death within 90 days in each arm, readmission rates were similar and   Within 90 days, there was one death in each arm. |
| Levine (2018) | During the care episode, home patients had fewer laboratory orders (median per admission: 6 vs. 19; p < 0.01) and less often received consultations (0% vs. 27%; p = 0.04). Home patients were more physically active (median minutes, 209 vs. 78; p < 0.01), with a trend toward more sleep. No adverse events occurred in home patients, one occurred in control patients.  There was a trend toward more hospital acquired disability in the control group: ADLs and IADLs were respectively worse at discharge in 9% and 18% of the control group vs. 0% and 0% of the home group |
| Levine (2020) | Compared with usual care patients, home patients had fewer laboratory orders (median per admission, 3 vs. 15), imaging studies (median, 14% vs. 44%), and consultations (median, 2% vs. 31%).   Home patients spent a smaller proportion of the day sedentary (median, 12% vs. 23%) or lying down (median, 18% vs. 55%) and were readmitted less frequently within 30 days (7% vs. 23%).  Nine percent of home patients and 15% of control patients had a safety event   Pain scores and frequency of delirium were similar between groups. No home patients and 10% of control patients received inappropriate medications. |
