# Supplementary material for: Transforming acute care: a scoping review on the effectiveness, safety and implementation challenges of Hospital-at-Home models
Source: BMJ Open. 2025 Aug 8;15(8):e098411. doi: 10.1136/bmjopen-2024-098411 (PMC12336546; doi:10.1136/bmjopen-2024-098411)
Supplement: Supplementary file 5 [file bmjopen-2024-098411.reviewer_comments.pdf]

PEER REVIEW HISTORY

BMJ Open publishes all reviews undertaken for accepted manuscripts. Reviewers are asked to complete a checklist review form and are provided with free text boxes to elaborate on their assessment. These free text comments are reproduced below.

ARTICLE DETAILS

Title (Provisional)

Transforming Acute Care: A scoping review on the effectiveness, safety and implementation challenges of Hospital-at-Home Models

Authors

Sultani, Khos; Smeulers, Marian; de Vries, Ralph; Zonderhuis, Barbara Maria; Nanayakkara, Prabath WB

VERSION 1 - REVIEW

**Reviewer**	**1**
**Name**	**Kanagala, Sai Gautham**
**Affiliation**	**Metropolitan Hospital Center, Internal Medicine**
**Date**	**19-Jan-2025**
**COI**	**None**

There have been prior systematic review and metanalysis which have been published in 2024 which higher number of studies, while your manuscript describes about limited number of studies. There is no new information talking about the effectiveness of the Hah models and neither about the future aspects where the Hah models need a change in the infrastructure discussed in the paper

---

**Reviewer**	**2**
**Name**	**Sloss, Elizabeth Ann**
**Affiliation**	**University of Utah Health**
**Date**	**06-Mar-2025**
**COI**	**None**

Overall, this scoping review of the literature is well-written, thorough, and timely. I have a few edits/suggestions that I hope will help to strengthen your manuscript:

Abstract/Methods: You state that a "systemic" literature search was conducted... do you mean systematic? Or scoping?

Main Text/Objectives: Your description of using the PCC approach contains redundant sentences and can be revised for clarity/brevity.

Main Text/Methods: I appreciate that you justify your rationale for including RCTs; however, I think that you need to acknowledge in the limitations section that you may have missed robust, pragmatic studies (matched-pairs design, for example) in your review given the very narrow selection criteria.

Supplement: Did you complete a PRISMA-ScR checklist? If so, I believe it is required to be included as a supplement.

Overall, I think that this article makes an informed and thoughtful contribution to the literature.

**Reviewer**	**3**
**Name**	**Maniaci, Michael**
**Affiliation**	**Mayo Clinic**
**Date**	**16-May-2025**
**COI**	**None**

This is a thoughtfully composed and well-organized scoping review focusing exclusively on randomized controlled trials evaluating hospital-at-home programs for acute care. The authors should be commended for addressing a clinically and strategically timely topic given the growing emphasis on decentralizing hospital care and relieving inpatient bed pressures, something i am passionate about. y limiting inclusion to RCTs, the review maximizes methodological rigor, elevating the reliability of conclusions on safety, cost-effectiveness, and patient outcomes.

I do believe just a few modifications would assist in making this manuscript stronger:

1. The abstract is succinct but underplays the gap in implementation science. Consider a final sentence like: “Despite favorable clinical outcomes, substantial implementation barriers remain unaddressed in current RCTs.” or something of the sort.

2. In some places (e.g., escalation rates), the text refers to “significant” or “comparable” outcomes without citing confidence intervals or p-values. Maybe clarify where possible to avoid overstating your conclusions.

3. use of "early avoidance" and "early discharge" --> As HsH is an inpatient model, i always want to make sure that these terms are either voided or used correctly . These terms are often blurred in this manuscript. Consider clearer categorization of which studies aligned with which model (e.g., Table 1 annotation or subgrouping results), pointing out transfer and substitution for ward care for HaH and hospital avoidance for outpatient care only.

4. Ona slightly same subject, I think terminology can be clarified in a few places. While the authors correctly cite the lack of a universal definition of HaH (although WHAHC did put one out), there is some inconsistency in terminology. For example, the interchangeable use of “HaH,” “GHHS,” and “substitutive care” could confuse readers unfamiliar with the field.

5. Don't know if it is possible from the RCT data (to back this up), but while correctly noting the dominance of COPD and CHF in the literature, this review would benefit from a clearer call to diversify HaH trials to include acute infections, post-operative care, and oncology --> maybe more of a call to action??

VERSION 1 - AUTHOR RESPONSE

Reviewer #1:

1. There have been prior systematic review and metanalysis which have been published in 2024 which higher number of studies, while your manuscript describes about limited number of studies. There is no new information talking about the effectiveness of the Hah models and neither about the future aspects where the Hah models need a change in the infrastructure discussed in the paper.

    Response:

    We thank the reviewer for this valuable comment. While we acknowledge that a number of systematic reviews and meta-analyses on Hospital-at-Home (HaH) have been published recently, including in 2024, our scoping review specifically focuses on randomized controlled trials (RCTs) evaluating admission avoidance and early supported discharge in acutely ill adults. This targeted scope, combined with strict inclusion criteria (e.g. excluding COVID-19-related studies and non-acute interventions), explains the lower number of included studies. Unlike previous broader reviews, our aim was to provide a focused overview of the highest level of evidence and to map the conditions under which HaH appears effective and safe.

    In addition, it does identify important knowledge gaps. A few examples are the underrepresentation of certain patient groups and lack of detailed reporting on implementation conditions which are essential for informing future clinical and infrastructural development of HaH programs. We have clarified this rationale and contribution more explicitly in both the introduction and conclusion sections of the manuscript.

Reviewer #2:

1. You state that a "systemic" literature search was conducted... do you mean systematic? Or scoping?

   Response: Thank you for pointing this out. We apologize for the typo. We conducted a systematic literature search as part of our scoping review methodology. We have corrected the term from "systemic" to "systematic" in the revised manuscript to avoid any confusion.

2. Your description of using the PCC approach contains redundant sentences and can be revised for clarity/brevity.

   Response: Thank you for your comment. We have revised the section describing the PCC framework to remove redundancy and improve clarity.

3. I appreciate that you justify your rationale for including RCTs; however, I think that you need to acknowledge in the limitations section that you may have missed robust, pragmatic studies (matched-pairs design, for example) in your review given the very narrow selection criteria.

   Response: Thank you for this valuable suggestion. We agree that by focusing exclusively on randomized controlled trials, we may have excluded high-quality, pragmatic studies, such as matched-pair design, that could provide meaningful insights into the effectiveness and implementation of HaH models. We have therefore added a sentence to the limitations section acknowledging this potential gap.

4. Did you complete a PRISMA-ScR checklist? If so, I believe it is required to be included as a supplement.

   Response: Yes, we completed the PRISMA-ScR checklist to ensure transparent and structured reporting of our scoping review. As suggested, we have now added the completed checklist as a supplementary file to the manuscript.

Reviewer #3:

1. The abstract is succinct but underplays the gap in implementation science. Consider a final sentence like: "Despite favorable clinical outcomes, substantial implementation barriers remain unaddressed in current RCTs." or something of the sort.

   Response: Thank you for this valuable suggestion. We agree that the abstract could better reflect the gap in implementation science. We have revised the final sentence of the abstract to: "Despite favorable clinical outcomes, substantial implementation barriers remain underexplored in current RCTs." This better emphasizes the need for future research focused on applicability and implementation strategies.

2. In some places (e.g., escalation rates), the text refers to "significant" or "comparable" outcomes without citing confidence intervals or p-values. Maybe clarify where possible to avoid overstating your conclusions.

   Response: Thank you for this valuable comment. We have critically reviewed the manuscript and clarified the use of terms such as "significant" and "comparable" where needed. To ensure transparency and avoid overstating our conclusions, we now explicitly refer readers to Table 3, where the reported p-values and confidence intervals, as provided in the original studies, are presented. This allows readers to assess the statistical strength of the findings themselves.

3. Use of "early avoidance" and "early discharge" --> As HsH is an inpatient model, i always want to make sure that these terms are either voided or used correctly . These terms are often blurred in this manuscript. Consider clearer categorization of which studies aligned with which model (e.g., Table 1 annotation or subgrouping results), pointing out transfer and substitution for ward care for HaH and hospital avoidance for outpatient care only.

   Response:
   Thank you for this observation. We have reviewed the manuscript to ensure consistent use of the terms *admission avoidance* and *early supported discharge* and added a dedicated "HaH model" column to Table 1 to indicate which model each study represents, thereby making the distinction explicit for readers.

4. On a slightly same subject, I think terminology can be clarified in a few places. While the authors correctly cite the lack of a universal definition of HaH (although WHAHC did put one out), there is some inconsistency in terminology. For example, the interchangeable use of "HaH," "GHHS," and "substitutive care" could confuse readers unfamiliar with the field.

   Response:

   Thank you for this comment. To minimise interpretation bias, we retained each study's original terminology (e.g., GHHS, substitutive care) only in Tables 2–4. In these tables results are reproduced verbatim, while throughout the narrative text we use the uniform term "Hospital-at-Home (HaH)." These alternate terms therefore appear exclusively in the tables and should be interpreted as synonymous with HaH.

5. Don't know if it is possible from the RCT data (to back this up), but while correctly noting the dominance of COPD and CHF in the literature, this review would benefit from a clearer call to diversify HaH trials to include acute infections, post-operative care, and oncology --> maybe more of a call to action??

   Response:

   Thank you for this valuable suggestion. While the included RCTs primarily focused on COPD and CHF, we agree that further research should broaden the scope of HaH interventions. In response, we have strengthened our call for future trials to explore underrepresented areas such as acute infections, post-operative care, and oncology, to better understand the broader applicability of HaH models.

VERSION 2 - REVIEW

**Reviewer**	**3**
**Name**	**Maniaci, Michael**
**Affiliation**	**Mayo Clinic**
**Date**	**03-Jul-2025**
**COI**	

The authors have done an excellent job of addressing both my concerns and the concerns of the other reviewers. I appreciate them for the hard work and dedication on this important subject.
